# Supplementary material for: The Salt Tolerance–Related Protein (STRP) Is a Positive Regulator of the Response to Salt Stress in Arabidopsis thaliana
Source: Plants (Basel). 2023 Apr 20;12(8):1704. doi: 10.3390/plants12081704 (PMC10145591; doi:10.3390/plants12081704)

Figure S1. PCR of CaMV 35S promoter confirms the integration of the transgenic construct. The integration of the construct in T2 lines was verified by genomic PCR with primers specific for the CaMV 35S promoter sequence (35S Fw 5′-GTCTCAGAAGACCAAAGGGC-3′; 35S Rev 5′-CCTCTCCAAATGAAATGAACTTCC-3′).

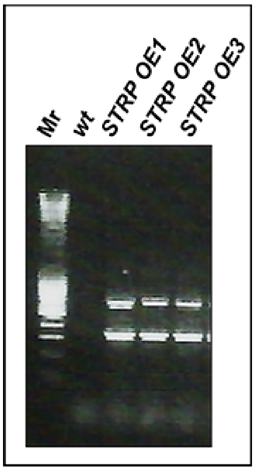

Figure S2. Representative confocal images of root and leaf tissues showing STRP-YFP overexpression. Representative root and leaf sections of *STRP* OE plants were imaged for GFP fluorescence by confocal microscopy with a laser scanning microscope (Olympus FV1000). Laser 488 nm (Argon) was used to detect the green fluorescence of YFP using a 20x objective.

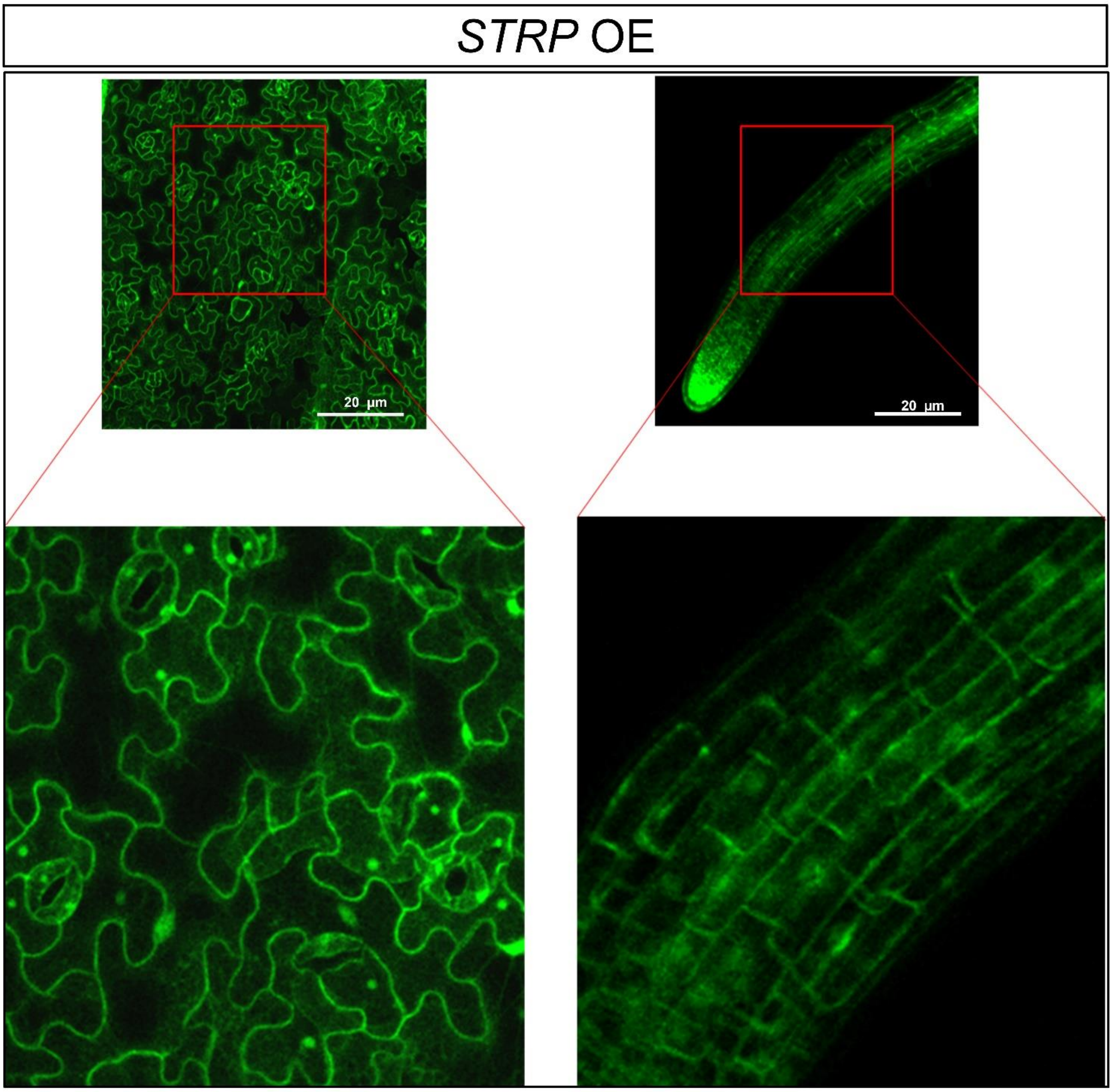

Figure S3. Full-length blot of STRP levels, with NaCl ranging from 50 mM to 150 mM, reported in the manuscript in Figure 1A.

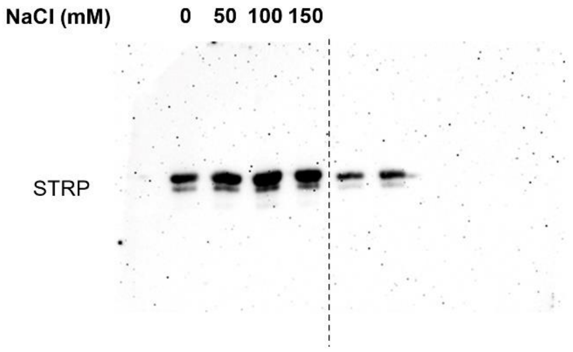

Figure S4. Full-length blot of the time-course experiment of STRP levels under 150 mM NaCl treatment, reported in the manuscript in Figure 1B.

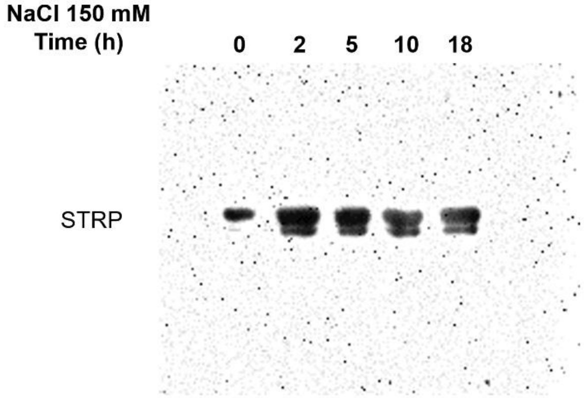

Figure S5. Full-length blot of MG132 experiment, reported in the manuscript in Figure 1D.

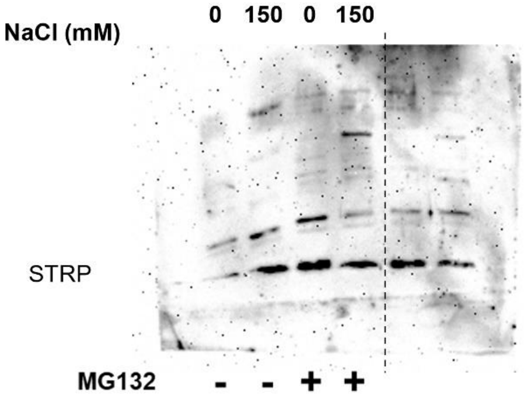

Figure S6. Full-length blots of *STRP* OE plants immunodecorated with anti-YFP and anti-STRP antibodies, reported in the manuscript in Figure 2B.

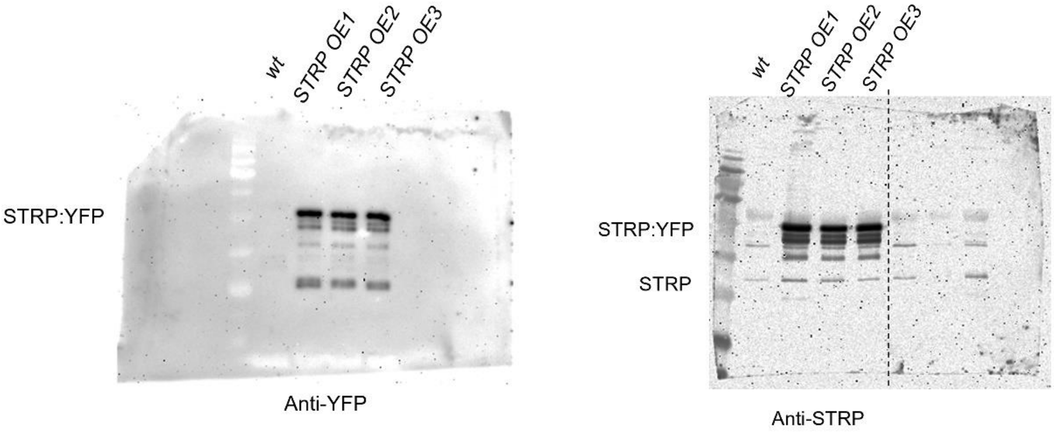

Figure S7. Full-length gel of genomic PCR for 35S integration in transgenic *STRP* OE lines, reported in the supplementary material in Figure S1.

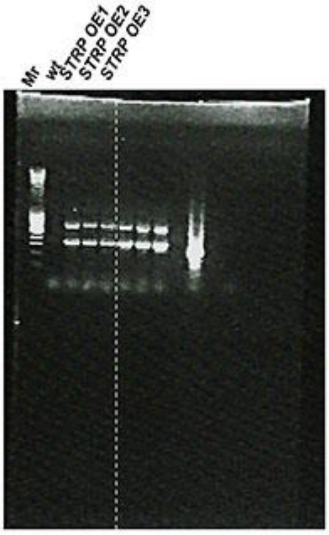

Supplement: Supplementary file 1 [file plants-12-01704-s001.zip › plants-2317666-supplementary.pdf]
